# Pulmonary Langerhans Cell Histiocytosis in the Elderly Smoker

**DOI:** 10.7759/cureus.10377

**Published:** 2020-09-11

**Authors:** Hafiz Muhammad Jeelani, Hamid Ehsan, Muhammad Mubbashir Sheikh, Adeel Riaz, Hafiz Mahboob

**Affiliations:** 1 Internal Medicine, Rosalind Franklin University of Medicine and Science, McHenry, USA; 2 Internal Medicine, MedStar Union Memorial Hospital, Baltimore, USA; 3 Biomedical Sciences/Biohazardous Threat Agents & Emerging Infectious Diseases, Georgetown University, Washington, DC, USA; 4 Oncology, Northwestern University Feinberg School of Medicine, Chicago, USA; 5 Anesthesiology and Critical Care, District Headquarter Hospital, Sahiwal, PAK; 6 Pulmonary and Critical Care Medicine, University of Nevada Las Vegas School of Medicine, Las Vegas, USA

**Keywords:** plch, lch, smoking, cd1a

## Abstract

Langerhans cell histiocytosis (LCH), formally referred to histiocytosis X, is a histiocytic disorder with unknown etiology. The pathogenesis is believed to originate from myeloid dendritic cells and is now considered an inflammatory myeloid neoplasm within the revised 2016 Histiocyte Society classification. Pulmonary Langerhans cell histiocytosis (PLCH) is a rare and isolated form of LCH with a strong affiliation with smoking in adults of 20-40 years of age. Characteristic CT chest and histologic findings are instrumental in the early recognition and management of a disease. We herein report a case of a Caucasian smoker female with a significant history of interstitial lung disease (ILD) presented with recurrent and progressive worsening dyspnea. History of ILD and recurring respiratory symptoms raised suspicion of PLCH. CT chest and pathological findings confirmed the diagnosis, and discontinuation of smoking resulted in favorable clinical outcomes.

## Introduction

Langerhans cell histiocytosis (LCH) is a rare systemic disorder of unknown origin that has a wide range of organ involvement. The pathological hallmark includes the accumulation of cluster of differentiation 1a (CD1a), langerin, and S100-positive dendritic cells of bone marrow origin in involved tissues resulting in the formation of eosinophilic granulomas, thus causing damage [1]. There is still a debate about the pathophysiology of whether LCH is immunoreactive or neoplastic as it shares features of both. Favoring immunoreactive, it resolves spontaneously, and on the other hand infiltration of abnormal cells of monoclonal origin favors the neoplastic process. The incidence of LCH is three to five cases per million per year in children younger than three years and one to two cases per million per year in adults [2]. Organ involvement also varies with age as in the young population most commonly involved organ is bones (~80%) followed by skin, pituitary gland, lungs, liver, and lymph nodes [3]. Isolated pulmonary Langerhans cell histiocytosis (PLCH) is the most common manifestation in the adult population. Smoking is the most common risk factor associated with PLCH, and the disease is often confounded by concurrent emphysema and restrictive lung diseases delaying the diagnosis. PLCH is a specific and rare cystic interstitial lung disease (ILD) with a variable presentation; it can resolve spontaneously, remain stable, or progress to advanced disease with the development of respiratory failure [4]. This article highlights the typical imaging and histological features of PLCH. As most of the reported cases are in young adults (20-40 years), presentation in the elderly in itself is a unique feature of PLCH.

## Case presentation

**Figure 1:**
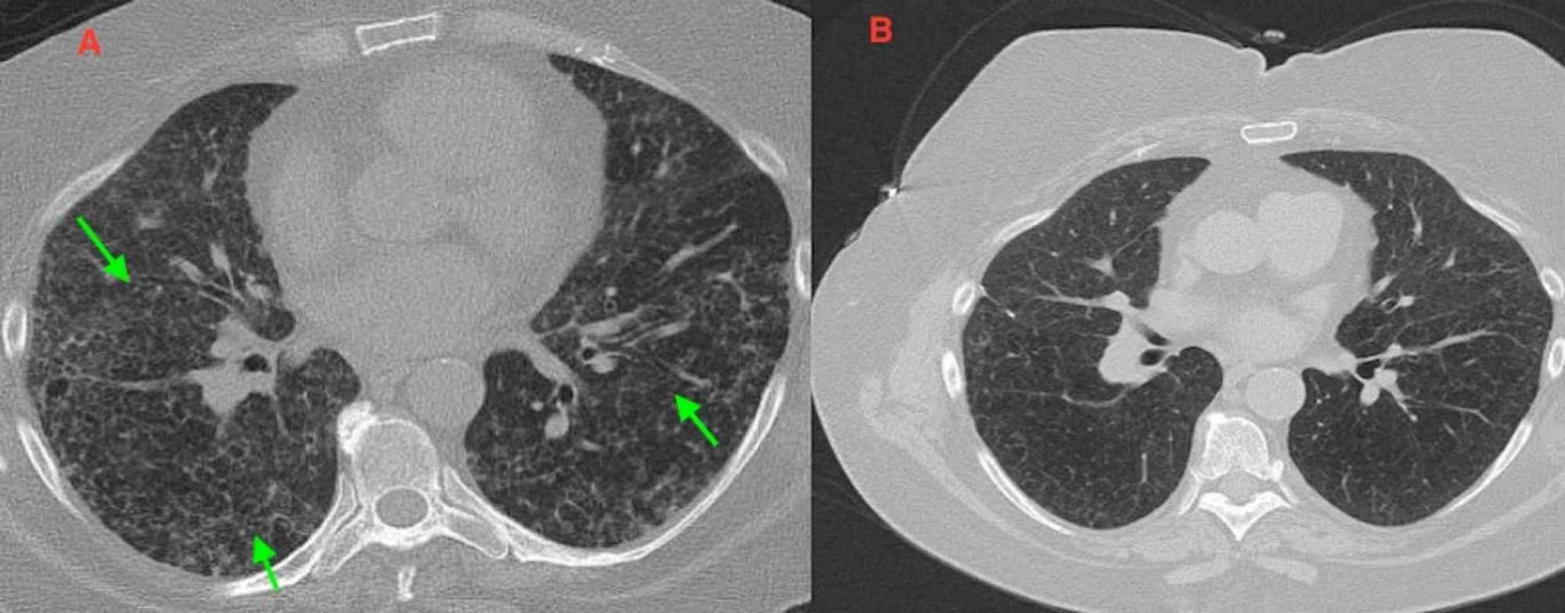
(A) CT chest shows diffuse reticular and nodular interstitial opacities, and ground-glass infiltrates, upper and mid lung predominant with lower lobes involvement (arrows). (B) Follow-up CT scan at three months shows interval improvement in reticulonodular and ground-glass infiltrates compared to (A).

A 63-year-old Caucasian female who smoked a pack of cigarettes per day for 45 years presented to the emergency department with a one-week history of shortness of breath. She denied fever, chills, hemoptysis, alcohol and illicit drug abuse, or any recent travel. Past medical history was significant for chronic obstructive pulmonary disease (COPD), type 2 diabetes mellitus, and ILD. Physical examination indicated a blood pressure of 159/89 mmHg, a heart rate of 74/min, a respiratory rate of 23/min, a temperature of 97.6˚F, and oxygen saturation of 74% on room air. Lung auscultation revealed bilateral diffuse crackles with no other objective abnormality. Complete blood count, basic metabolic panel, liver function tests were unremarkable. Pulmonary function tests (PFTs) demonstrated a restrictive pattern with a marked reduction in diffusing capacity for carbon monoxide (DLCO 39%). CT scan of the chest showed upper and mid lung predominant diffuse reticular and nodular interstitial opacities, and ground-glass infiltrates, worsened from prior imaging (Figure 1).

**Figure 2:**
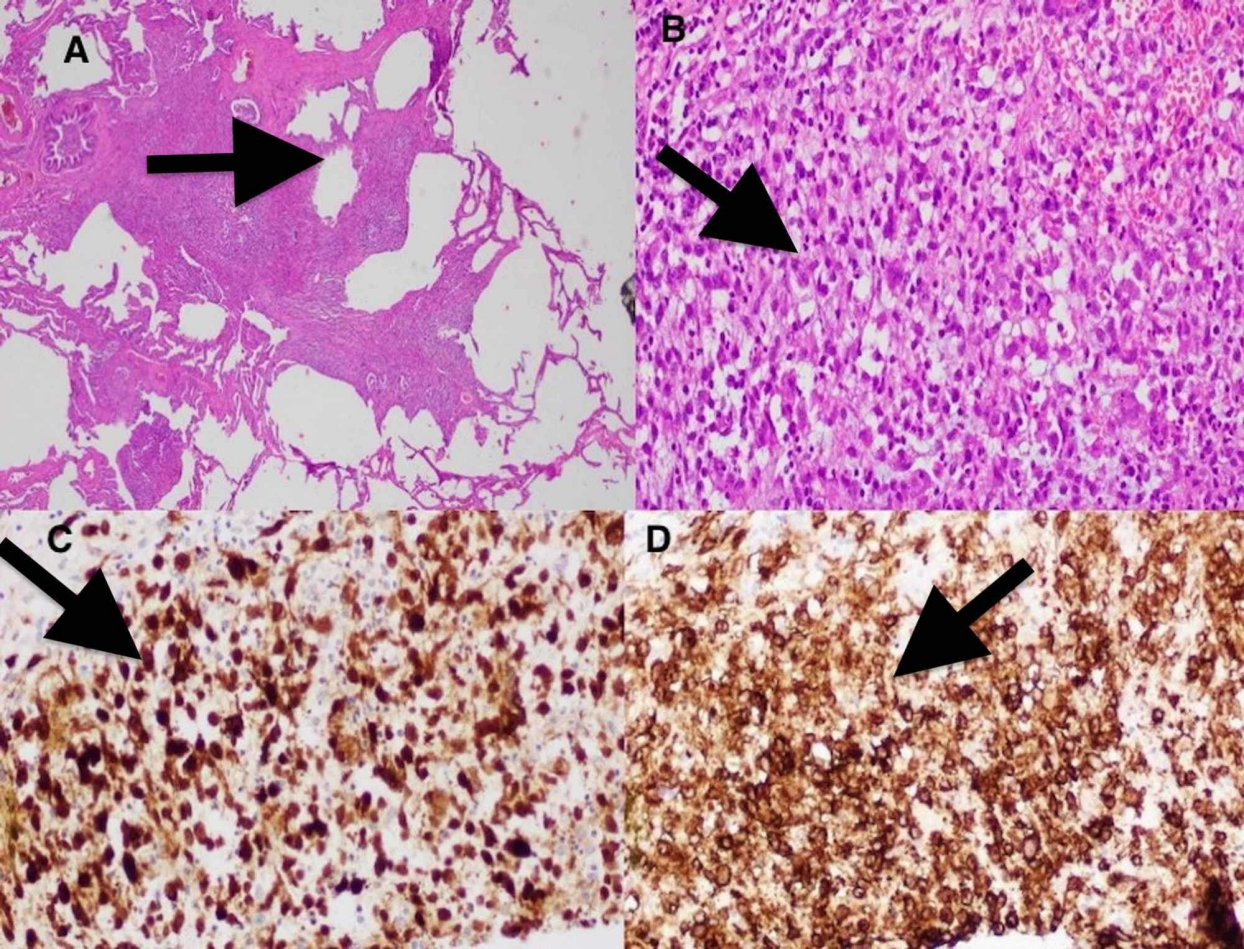
(A) H&E staining of biopsied specimen at low magnification showing stellate nodules and cysts characteristic of LCH. (B) Higher magnification showing histiocytes (Langerhans cells) with crumpled tissue paper nuclear contour. Langerhans cell showing positive staining for S-100 (C) and CD1a (D). LHC: Langerhans cell histiocytosis, H&E: hematoxylin and eosin, CD1a: cluster of differentiation 1a

After noting a progression of ILD, a decision was made to perform a video-assisted thoracoscopic surgery (VATS) with wedge resection of the right upper, middle, and lower lobe lobes. Hematoxylin-eosin staining showed a large number of typical Langerhans cells in the lung tissue, with crumpled tissue paper nuclear contours. Immunohistochemical analysis revealed positivity for CD1a and S100 (Figure 2).

Initially, the patient was managed as a case of exacerbation of COPD and started on intravenous (IV) steroids and supplemental oxygen to maintain an oxygen saturation of 92%. She also received a single dose of Lasix® 40 mg for suspicion of congestive heart failure (CHF). There was also a concern for hypersensitivity pneumonitis, so a rheumatologic panel, including antinuclear antibody (ANA), rheumatoid factor (RF), angiotensin-converting enzyme (ACE) levels, and a hypersensitivity pneumonitis panel, was checked, which came back negative. After imaging and pathologic confirmation, the final diagnosis of PLCH was made. Her shortness of breath improved remarkably throughout the admission. She was discharged on oral prednisone 0.5 mg/kg, supplemental oxygenation, and strongly advised to quit smoking which she adhered to.

Outpatient follow-up at three months showed a significant improvement in the patient's clinical status. Chest CT performed showed an interval improvement predominantly in upper lobe reticular ILD, which was consistent with the improvement of LCH (Figure 1B). The patient continued to follow with pulmonary medicine and has significant improvement in the quality of life by just smoking cessation and three months of steroids use.

## Discussion

PLCH has a variable clinical presentation from being minimally symptomatic to progressive respiratory failure. The disease's initial stage is characterized by predominant upper and middle lobe reticulonodular infiltrates with relative sparing of lung bases. However, diffuse involvement is seen in older patients. Reduced diffusion capacity is the most frequently observed abnormality in PFTs [5]. In older patients, comorbidities can further confound the clinical and radiological presentation, and diagnosis can be delayed, as in our patient, who was initially treated as a case of COPD exacerbation but was later found to have PLCH via imaging and biopsy findings. 

Typical clinical and radiological presentation in young adults precludes tissue diagnosis. However, atypical presentation warrants biopsy [5]. Positron emission tomography (PET) scan is useful for identifying disease activity, extrapulmonary sites, and therapy response in established cases. It can offer potential benefits in patients who cannot undergo biopsy [6]. The factors associated with the poor prognosis of PLCH include older age, obstructive lung disease pattern, markedly decreasing DLCO, multisystemic involvement, and active smoking [4]. The primary cause of death is respiratory failure secondary to the development of pulmonary hypertension over the course of a disease. Other associated complications include bronchogenic carcinoma (double risk in the presence of active smoking), recurrent spontaneous pneumothorax/pneumothoraces (15%-25%), and hemoptysis (13%).

To date, there are no standard therapies for the adult population. Smoking cessation is considered the only effective strategy in PLCH followed by corticosteroids. Other treatment options include the use of cytotoxic drugs (methotrexate, cyclophosphamide) and cladribine (2-chlorodeoxyadenosine), a purine nucleoside analog, that have shown beneficial effects in severe PLCH [7]. Currently, an ongoing phase II trial is evaluating the efficacy and tolerability of cladribine (ECLA) as monotherapy in symptomatic patients with PLHC and impairment of lung functions (NCT01473797) [8]. Patients will be receiving subcutaneous injections of cladribine 0.1 mg/kg/day for five days as a single course per month for four months. The primary outcome of this study is to assess the cumulated incidence of response to treatment. The response will be defined as ≥10% improvement of forced vital capacity (FVC) or post-bronchodilator forced expiratory volume in one second (FEV1) assessed after six months of treatment. Chemotherapeutic agents targeting against BRAF and MAPK-21 mutations reported to be effective as well in PLHC patients [9]. In the case of aggressive PLCH, inadequate response to therapy or with the development of severe pulmonary hypertension, lung transplant should be considered, as later is associated with a worse prognosis [10].

## Conclusions

In elderly patients with significant smoking history, PLCH can have non-specific clinical, PFTs, and radiologic presentations that can delay the diagnosis. A high index of suspicion for PLCH in this age group is prudent, as timely intervention can halt the disease progression and improve clinical outcomes. However, long-term follow-up is still imperative even after achieving clinical improvement to avoid future complications associated with PLCH.
